# Supplementary material for: Copy number variation of the restorer Rf4 underlies human selection of three-line hybrid rice breeding
Source: Nat Commun. 2023 Nov 13;14:7333. doi: 10.1038/s41467-023-43009-4 (PMC10643609; doi:10.1038/s41467-023-43009-4)
Supplement: Supplementary file 1 — Supplementary Information [file 41467_2023_43009_MOESM1_ESM.pdf]

# Supplementary Information

## Copy number variation of the restorer *Rf4* underlies human selection of three-line hybrid rice breeding

*Zhao et al.*

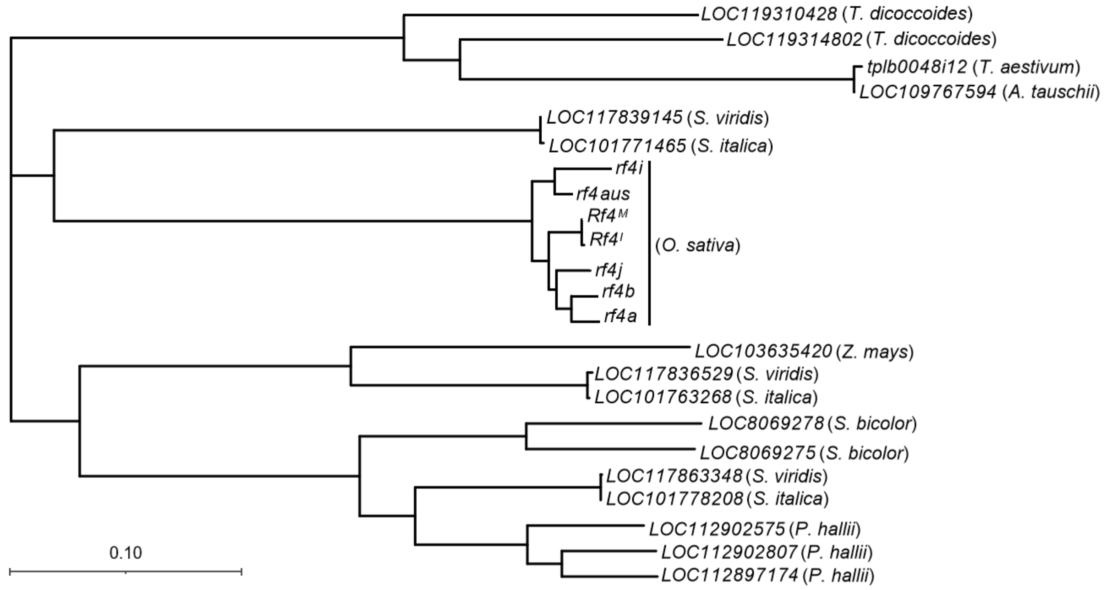


**Supplementary Fig. 1. Phylogenetic tree of *Rf4* orthologs in the Poaceae.**
The putative orthologs were identified by searching the Poaceae sequences in the NCBI database using the coding sequence of *Rf4* as a query. These orthologs were then used to construct a phylogenetic tree. The bootstrap values indicate the percentage of support from 1,000 sampled trees. The scale bar indicates the number of nucleotide substitutions per site.

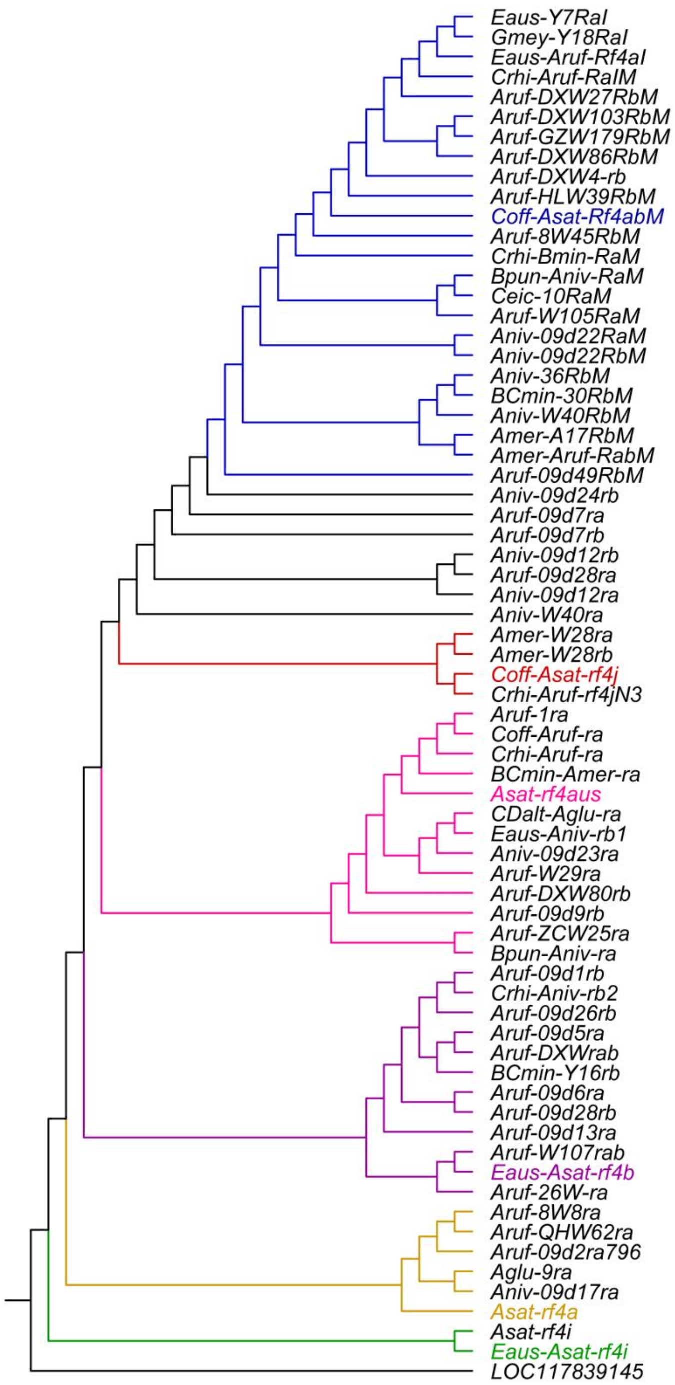


**Supplementary Fig. 2. Phylogenetic relationships of *Rf4* homologues in the *Oryza* genus.**

Maximum likelihood phylogenetic tree of the nucleotide sequences of *Rf4* and *rf4*. *LOC117839145* from *Setaria viridis* served as an outgroup; 1,000 rapid bootstrap inferences were performed. Blue branches indicate *Rf4*, green branches indicate *rf4i*, yellow branches indicate *rf4a,* purple branches indicate *rf4b*, reddish violet branches indicate *rf4aus*, red branches indicate *rf4j*, and homologs. The uppercase letter before the "-" indicates the genome type of wild rice from AA to GG; the three lowercase letters represent the abbreviated genus names.

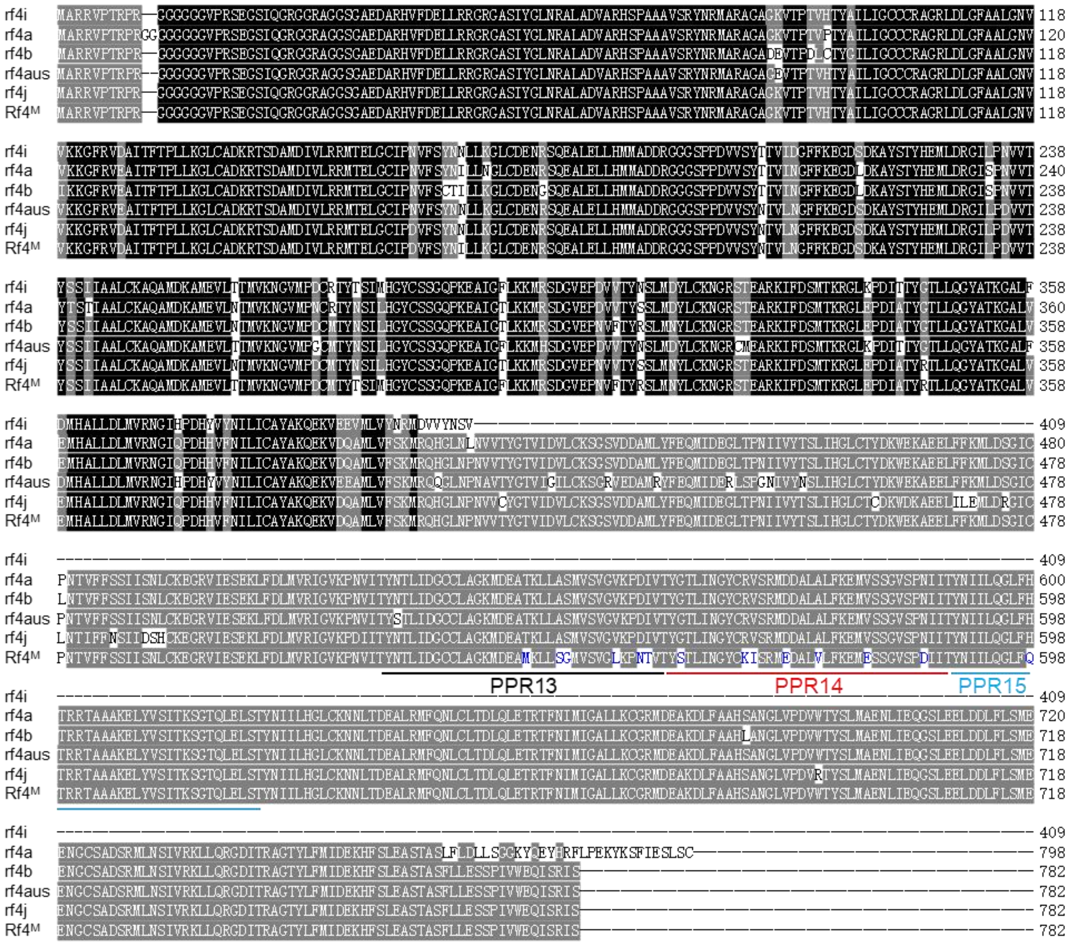


**Supplementary Fig. 3. Sequence alignment of Rf4 and rf4 proteins from Asian rice cultivars.**

Blue letters indicate the 14 amino acids in PPR13, PPR14, and PPR15 that differ between rf4 and Rf4 proteins.

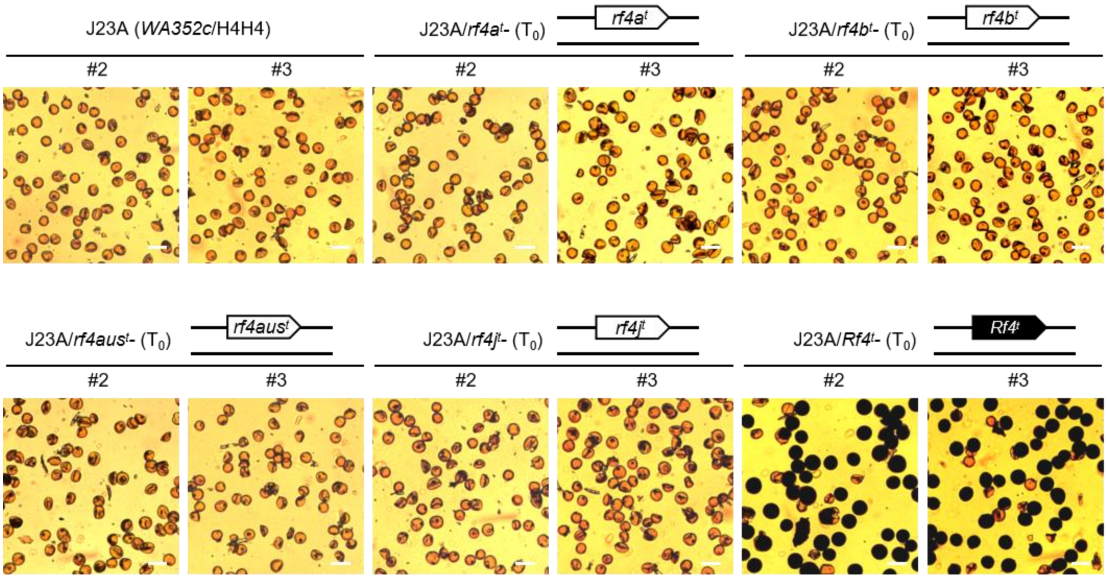


**Supplementary Fig. 4. Functional characterization of *Rf4* haplotypes in cultivars.** Pollen fertility of two biological replicates of five transgenic lines carrying *rf4a*, *rf4b*, *rf4aus*, *rf4j* and *Rf4* in Jin23A (a CMS-WA line) background. Only *Rf4*$^t$ lines can restore the fertility of Jin23A in the transgenic plants. Scale bars: 50 µm.

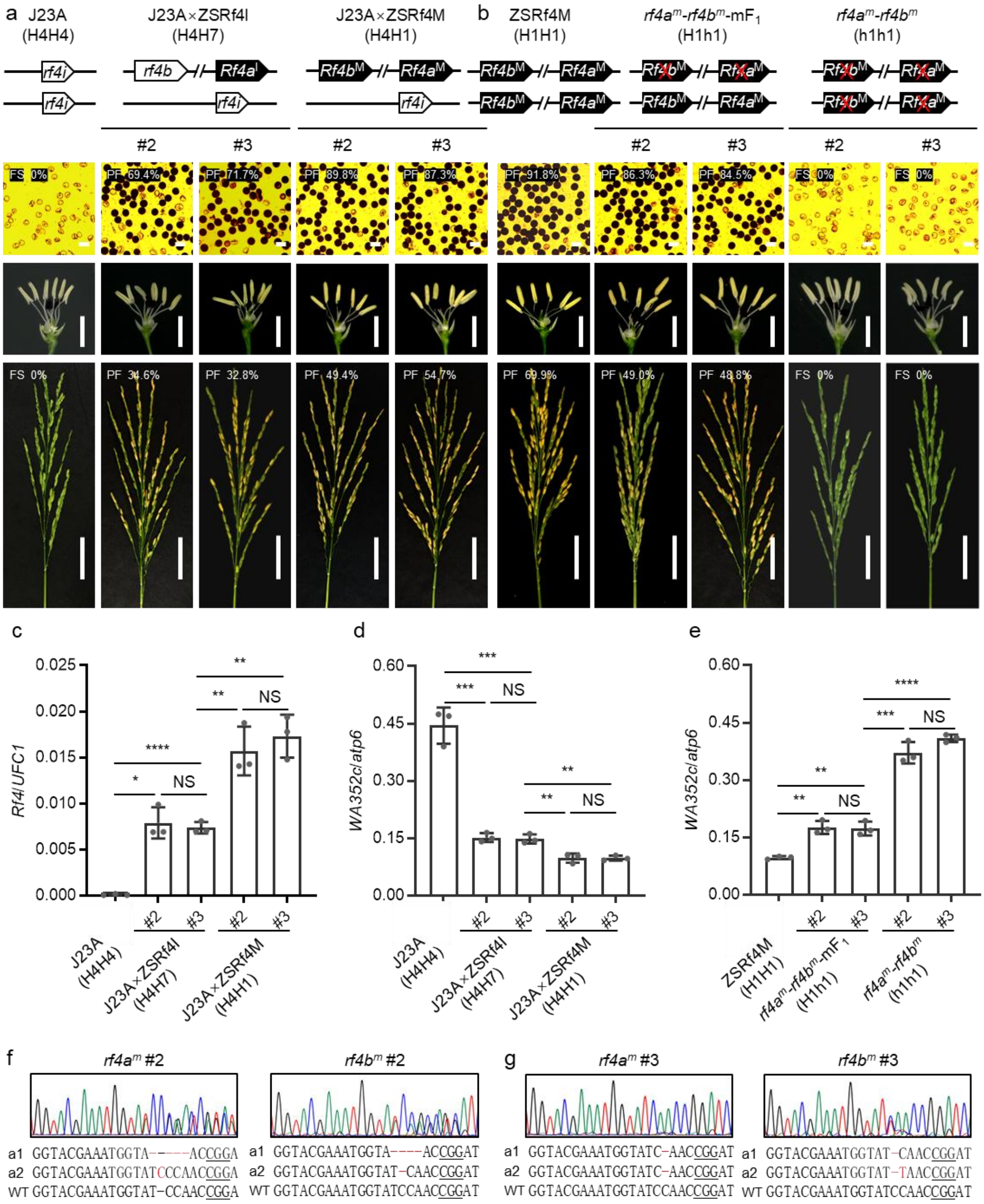


**Supplementary Fig. 5. Validation of dosage effect of *Rf4* using *Rf4* near-isogenic lines.**

**a** and **b** Pollen viability (upper panels), anther phenotype (middle panels), and seed setting rate (lower panels) of two biological replicates of Jin23A×ZSRf4I, Jin23A×ZSRf4M and *rf4a*$^{m}$-*rf4b*$^{m}$-mF$_{1}$/*rf4a*$^{m}$-*rf4b*$^{m}$ mutant lines in the ZSRf4M background. Red "×" indicates the non-functional *rf4a*/*b*$^{m}$ after knockout of *Rf4a*/*b*$^{M}$. Scale bars: 50 µm in the upper panels, 1 cm in the middle panels, and 5 cm in the lower panels. **c**-**e** Transcript levels of *Rf4* (**c**) and *WA352c* (**d**, **e**) in different lines. *UFC1* and *atp6* served as internal reference of nuclear and mitochondrial gene, respectively. Data are shown as mean ± SD, $n$ = 3 biological replicates. Significant differences between two samples were determined by two-tailed Student's $t$-test (*$P < 0.05$, **$P < 0.01$,

***$P < 0.001$, ****$P < 0.0001$, and NS represents No Significance). Source data are provided in the Source Data file. **f** and **g** Sequencing of two independent *Rf4a/b*$^{M}$-knockout plants. The underlined bases show protospacer adjacent motifs (PAMs). The positions highlighted in red indicate the targeted mutations.

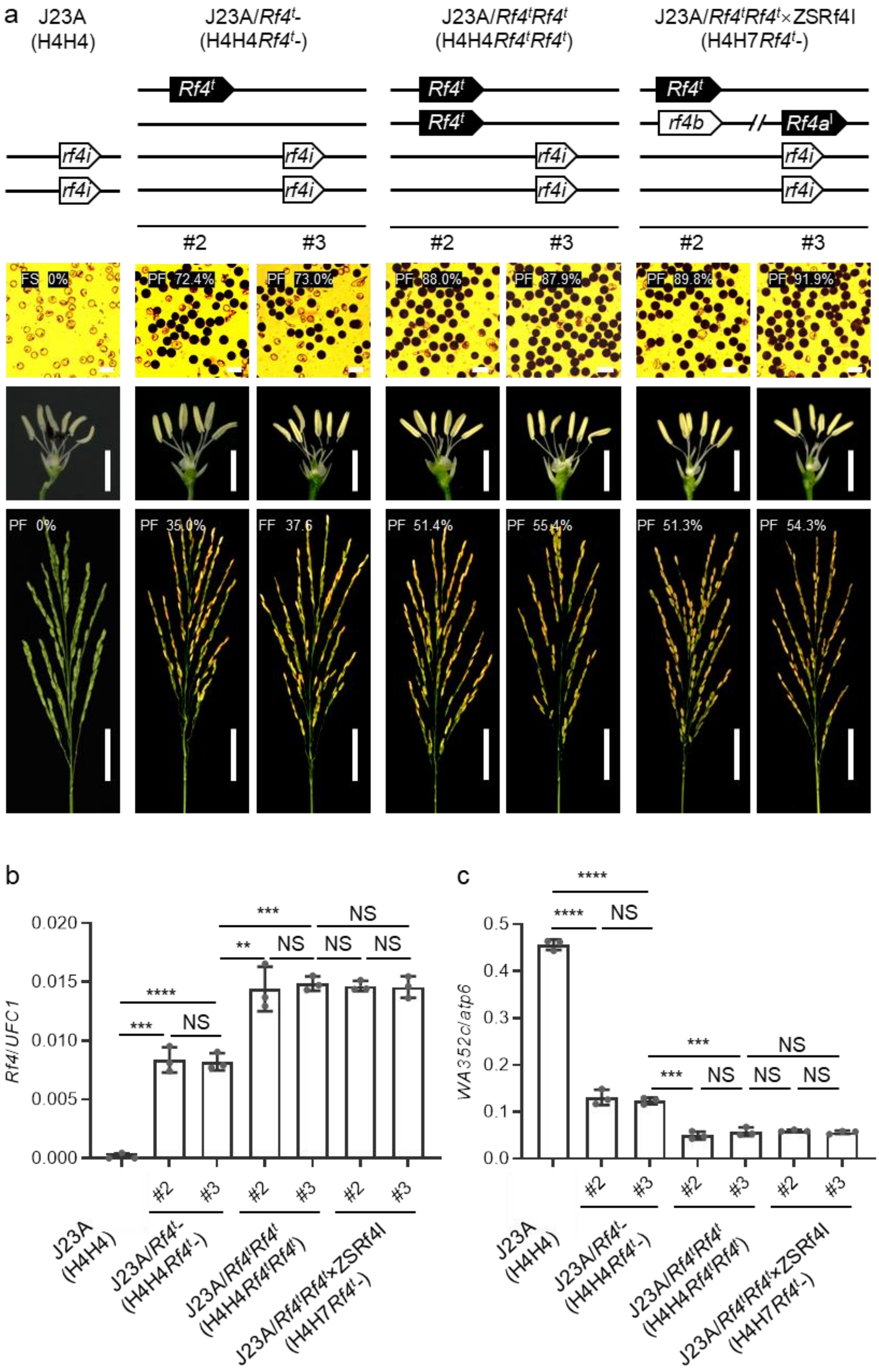


**Supplementary Fig. 6. Confirmation of the dosage effect of *Rf4* in complementary lines.**

**a** Pollen fertility (upper panels), anther phenotype (middle panels) and seed setting rate

(lower panels) of two biological replicates of Jin23A/*Rf4*$^{t}$-, Jin23A/*Rf4*$^{t}$*Rf4*$^{t}$, and Jin23A/*Rf4*$^{t}$*Rf4*$^{t}$×ZSRf4I. Scale bars: 50 μm in the upper panels, 1 cm in the middle panels, and 5 cm in the lower panels. **b**, **c** Transcript levels of *Rf4* (**b**) and *WA352c* (**c**) in different lines. "*Rf4*$^{t}$" indicates the *Rf4* transgene, "*Rf4*$^{t}$-" indicates transgenic hemizygotes. Data are shown as mean ± SD, $n$ = 3 biological replicates. Significant differences between two samples were determined by two-tailed Student's $t$-test (**$P < 0.01$, ***$P < 0.001$, ****$P < 0.0001$, and NS represents No Significance). Source data are provided in the Source Data file.

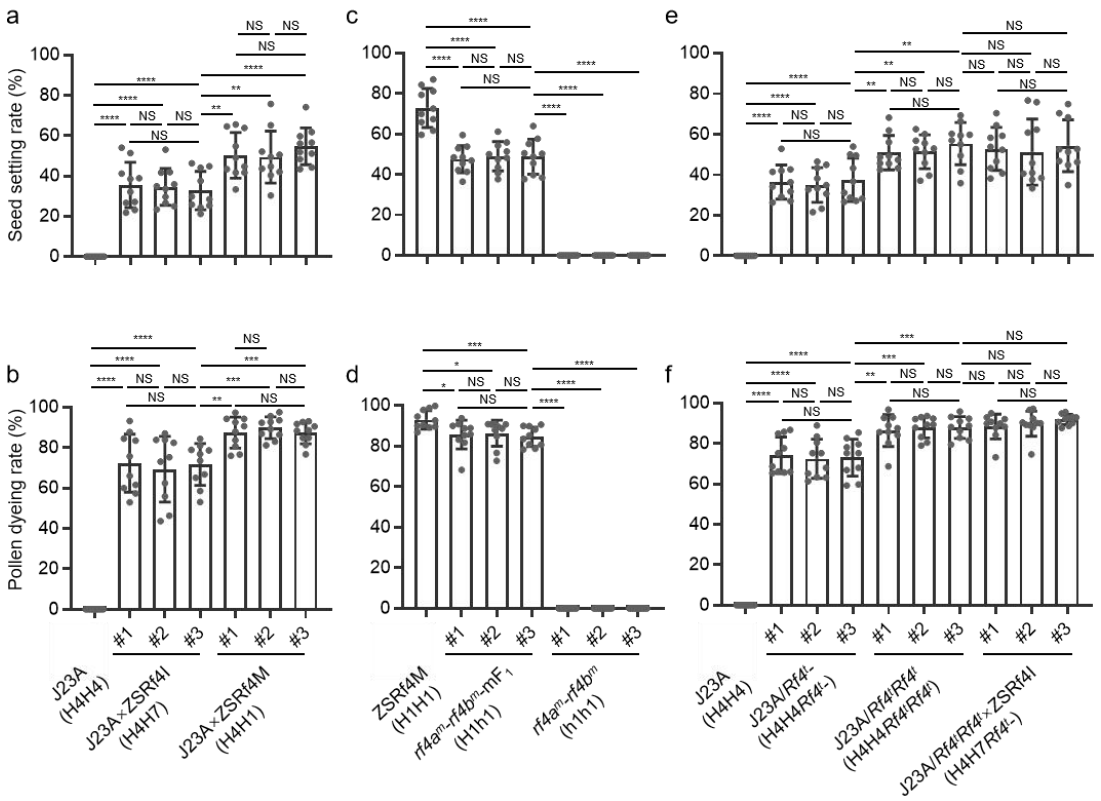


**Supplementary Fig. 7. Statistical analysis of seed setting rate and pollen dyeing rate in different *Rf4*-containing lines.**

**a**, **c** and **e** show seed setting rate, **b**, **d** and **f** show pollen dyeing rate (viability). Data are shown as mean ± SD, $n$ = 10 biological replicates. Significant differences between two samples were determined by two-tailed Student's $t$-test (*$P < 0.05$, **$P < 0.01$, ***$P < 0.001$, ****$P < 0.0001$, and NS represents No Significance). Source data are provided in the Source Data file.

**Supplementary Table 1. Primers used in this study.**

| Primer name | Primer sequence (5′-3′) | Purpose |
|---|---|---|
| STI-R/rf4-F1 | gcctggctccacgctccgagtGGGAGTGAGGGATAAAAGCATTGC | Amplification and detection of the *Rf4* locus |
| STI-R/rf4a-R | gcctggctccacgctccgagtCGCACTTTTGGGTCAAATTGACGTC | |
| STI-R/rf4b-R | gcctggctccacgctccgagtATCCACGTCGAGCTCTACGACCACA | |
| rf4i-F2 | TCACTCGCGCATCAAACGT | |
| rf4i-R | CGCTGAAGTATACGATTTTGCGT | |
| R/rf4-seq-F1 | CATCAAACGTGAGGGCACCC | Sequencing of PCR products |
| R/rf4-seq-F2 | AGTGCTCCGCAGAATGACCG | |
| R/rf4-seq-F3 | AATATGTGCATACGCTAAAC | |
| R/rf4-seq-F4 | ACACAGCTTGAACTTAGCAC | |
| rf4i-seq-F1 | CATCAAACGTGAGGGCACCC | |
| rf4i-seq-F2 | CACATGATGGCTGATGAT | |
| Rf4-262 bp-F | CTTTAGTTCAATAATTAGCGATCT | Molecular markers of different *Rf4* haplotypes |
| Rf4-262 bp-R | GCGTCTTCCATCCTACTAGTTTTG | |
| Copy-a-332 bp-F | GACGGTTACCGCACTACCGCA | |
| Copy-a-332 bp-R | CACGCACACTCATCCCTATGAAAGT | |
| Copy-b-282 bp-F | TTCGGTCGGTAGTGGGAATA | |
| Copy-b-282 bp-R | CCGTCGAGCTCTCCGACCACA | |
| rf4a-446 bp-F | GTCATGCCTAATTGCAGGACG | |
| rf4a-446 bp-R | CCATAGGTCACTACATTCA | |
| rf4b-197 bp-F | GCAGAATGACCGAGCTCGGC | |
| rf4b-197 bp-R | CAGATCCCCCTCTGTGAAGAAT | |
| rf4i-358 bp-F | CAGCCGAAAGAGGCTATTGGATT | |
| rf4i-358 bp-R | CAACGTCCATCCTGTTAT | |
| rf4j-372 bp-F | TGGATTGAATCCGAATGTAGAGTG | |
| rf4j-372 bp-R | GCTAAGCAGCATCCATCGATGAGT | |
| rf4aus-351 bp-F | CTATGACCAAGAGGGGCCTT | |
| rf4aus-351 bp-R | CAATGTTGCCAGGGATTAGTCT | |
| Actin1-F | GGAATGGAAGCTGCGGGTA | |
| Actin1-R | ACCACAGGTAGCAATAGGTA | |
| gRf4-NP-F | AACAGCTATGACATGATTACGAATTCGAAGAACCATTGATGCTGGAAC | Transgenic and segregation analysis |
| gRf4-NP-R | CCAATTCACACTTGTA**ggatcc**GCCTGCCCCCGCGGGCGCCA | |
| gRf4/rf4-F | CTGGCGCCCGCGGGGGCAGGCATGGCGCGCCGCGTCCCTACCCGCC | |
| gRf4/rf4-R | AATTCACACTTGTA**ggatcc**CTACGATATTCTTGATATTTGC | |
| gRf4-check-F | TTAGCTCACTCATTAGGCAC | |
| gRf4-check-R | CTGCTAGCTTCTTCTGCACA | |
| Hpt-F | ATTTGTGTACGCCCGACAGT | |
| Hpt-R | GTGCTTGACATTGGGGAGTT | |
| 1-gRf4-T-DNA-F1 | CCAAACGGCCAAAGCTTCAT | |
| 1-gRf4-T-DNA-R1 | TTCAAGAATTCAAGTACTCC | |
| 2-gRf4-T-DNA-F1 | GTCTATCGTGGATGAAACGT | |
| 2-gRf4-T-DNA-R1 | GTAGTGGGTATTGTGAAAGG | |
| 3-gRf4-T-DNA-F1 | CGCGTGGGACCCATCTGTCAT | |
| 3-gRf4-T-DNA-R1 | CGGTAGGGGTGACGCGATGT | |
| Hpt-F2 | ATCGTCCGATCCGGAGCCGGGAC | |
| 1-gRf4-T-DNA-R2 | TGCCGCTGCCACTACTATTT | |
| 2-gRf4-T-DNA-R2 | GTGCCAAAGTTAGTGACAACA | |
| 3-gRf4-T-DNA-R2 | GTGAGGTGAGGCGGGATCA | |
| WA352c-F | ACAAGCAAGGGTTGTAGGTCCATT | |
| WA352c-R | ACCTATTATTCGTAGGCGGTACGTA | |
| WA352c-seq-F | ATAAGGGGGAGTAGTAGGAG | |
| Rf4-qRT-F | CCTGATTGCATGACATATAC | Gene expression analysis by qRT-PCR |
| Rf4-qRT-R | CATCAGTGATCTGTAAGTAAA | |
| UFC1-qRT-F | GATGGCAAGACCCACAAG | |
| UFC1-qRT-R | TCCCGAACCTTGGGCAGT | |
| WA352c-qRT-F | CATGCAAATGTCCCGGATTCAAGC | |
| WA352c-qRT-R | GTAAGCGGACTCTTTCGACCAAG | |
| atp6-qRT-F | GGCATTACGATCGTTGGATTTC | |
| atp6-qRT-R | TTGATGGAGATTTATAGCATCATTC | |
| Rf4-KO-gRT1 | GTACGAAATGGTATCCAACgttttagagctagaaat | Editing target of *Rf4*-KO and genotype analysis of the targeted mutations |
| Rf4-KO-OsU6aT1 | GTTGGATACCATTTCGTACCggcagccaagccagca | |
| STI-R/rf4-F1 | gcctggctccacgctccgagtGGGAGTGAGGGATAAAAGCATTGC | |
| STI-R/rf4a-R | gcctggctccacgctccgagtCGCACTTTTGGGTCAAATTGACGTC | |
| STI-R/rf4b-R | gcctggctccacgctccgagtATCCACGTCGAGCTCTACGACCACA | |
| Rf4-KO-Seq-F | GCGCAGTGATGGTGTCGAA | |

Note: The primers used for STI PCR; 5′-tags are shown in red, the 5′-tags for Gibson Assembly cloning are underlined, and restriction enzyme sites are shown in bold.

**Supplementary Table 2. Genetic analysis of ZSRf4M-*Rf4*-KO plants.**

| Line | Target of $Rf4a^{M}$ | Target of $Rf4b^{M}$ | Genotype | Phenotype |
|---|---|---|---|---|
| Ref. | GGTACGAAATGGTATCC-AACCGG<br>GGTACGAAATGGTATCC-AACCGG | GGTACGAAATGGTATCCAACCGG<br>GGTACGAAATGGTATCCAACCGG | WT | FF |
| #1 | GGTACGAAATGGTA-----ACCGG<br>GGTACGAAATGGTA-----ACCGG | GGTACGAAATGGTATC-AACCGG<br>GGTACGAAATGGTATC-AACCGG | Homozygote | FS |
| #2 | GGTACGAAATGGTA-----ACCGG<br>GGTACGAAATGGTATCCCAACCGG | GGTACGAAATGGTATC----CGG<br>GGTACGAAATGGTAA-CAACCGG | Bi-allelic | FS |
| #3 | GGTACGAAATGGTATC--AACCGG<br>GGTACGAAATGGTATC--AACCGG | GGTACGAAATGGTATT-AACCGG<br>GGTACGAAATGGTATC-AACCGG | Bi-allelic | FS |
| #4 | GGTACGAAATGGTATC-----CGG<br>GGTACGAAATGGTAA-C-AACCGG | GGTACGAAATGGTA----ACCGG<br>GGTACGAAATGGTA----ACCGG | Bi-allelic | FS |
| #5 | GGTACGAAATGGTATCC-AACCGG<br>GGTACGAAATGGTATCC-AACCGG | GGTACGAAATGGTATCCAACCGG<br>GGTACGAAATGGTATCCAACCGG | WT | FF |

Note: The PAMs (CGG) are underlined. The positions highlighted in red indicate the targeted mutations. 'Homozygote' indicates the line is homozygous mutant for one allele, 'Bi-allelic' indicates the heterozygous line carrying two different mutant alleles. 'FS' and 'FF' indicate full sterility and full fertility of pollen, respectively.

**Supplementary Table 3. Haplotypes of *Rf4* in major restorer lines and the planting areas (hectares, ha) of the bred hybrid rice varieties in China.**

| Restorer line | Haplotype | Planting area (ha) |
|---|---|---|
| MH63 | H1 ($Rf4a^{M}$-$Rf4b^{M}$) | 83,912,667 |
| Ce64-7 | H1 ($Rf4a^{M}$-$Rf4b^{M}$) | 29,987,333 |
| CDR22 | H1 ($Rf4a^{M}$-$Rf4b^{M}$) | 9,706,667 |
| FuHui838 | H1 ($Rf4a^{M}$-$Rf4b^{M}$) | 9,297,333 |
| MH86 | H1 ($Rf4a^{M}$-$Rf4b^{M}$) | 2,584,000 |
| ChengHui727 | H1 ($Rf4a^{M}$-$Rf4b^{M}$) | 510,667 |
| Miyang46 | H7 ($Rf4a^{I}$-*rf4b*) | 14,242,000 |
| Gui99 | H7 ($Rf4a^{I}$-*rf4b*) | 10,237,333 |
| IR24 | H7 ($Rf4a^{I}$-*rf4b*) | 10,152,000 |
| R402 | H7 ($Rf4a^{I}$-*rf4b*) | 7,896,667 |
| Shuhui527 | H7 ($Rf4a^{I}$-*rf4b*) | 7,141,333 |
| Huazhan | H7 ($Rf4a^{I}$-*rf4b*) | 6,814,000 |
| Guanghui998 | H7 ($Rf4a^{I}$-*rf4b*) | 4,071,333 |
| Minhui3301 | H7 ($Rf4a^{I}$-*rf4b*) | 711,333 |
| XianHui207 | H8 (*rf4a*-$Rf4b^{M}$) | 7,507,333 |
| MianHui725 | H6 ($Rf4b^{M}$) | 9,242,000 |

Notes The data are from the China Rice Data Center, 1982 to 2019 (https://www.ricedata.cn/).
