# Supplementary material for: Copy number variation of the restorer Rf4 underlies human selection of three-line hybrid rice breeding
Source: Nat Commun. 2023 Nov 13;14:7333. doi: 10.1038/s41467-023-43009-4 (PMC10643609; doi:10.1038/s41467-023-43009-4)
Supplement: Supplementary file 3 — Description of Additional Supplementary Files [file 41467_2023_43009_MOESM3_ESM.pdf]

Description of Additional Supplementary Files

File Name: Supplementary Data 1
Discription: Similarity of 7.5 kb upstream of the start codon, and 1.5 kb downstream of the stop codon of *Rf4a*^{M} and *Rf4b*^{M}.

File Name: Supplementary Data 2
Discription: Cultivated rice accessions used for sequence analysis.

File Name: Supplementary Data 3
Discription: Wild rice accessions used for sequence analysis.

File Name: Supplementary Data 4
Discription: Landrace accessions used for sequence analysis.

File Name: Supplementary Data 5
Discription: 68 variants of *Rf4* locus

File Name: Supplementary Data 6
Discription: Nucleotide similarity of *Rf4*^{M} variants vs other homologs of Poaceae.

File Name: Supplementary Data 7
Discription: Protein similarity of *Rf4*^{M} variants vs other homologs of Poaceae.
