# Supplementary material for: Copy number variation of the restorer Rf4 underlies human selection of three-line hybrid rice breeding
Source: Nat Commun. 2023 Nov 13;14:7333. doi: 10.1038/s41467-023-43009-4 (PMC10643609; doi:10.1038/s41467-023-43009-4)
Supplement: Supplementary file 11 — Reporting Summary [file 41467_2023_43009_MOESM11_ESM.pdf]

nature portfolio

Corresponding author(s): Letian Chen & Yongyao Xie

Last updated by author(s): Oct 23, 2023

Reporting Summary

Nature Portfolio wishes to improve the reproducibility of the work that we publish. This form provides structure for consistency and transparency in reporting. For further information on Nature Portfolio policies, see our Editorial Policies and the Editorial Policy Checklist.

Statistics

For all statistical analyses, confirm that the following items are present in the figure legend, table legend, main text, or Methods section.

n/a	Confirmed	
☐	☒	The exact sample size (*n*) for each experimental group/condition, given as a discrete number and unit of measurement
☐	☒	A statement on whether measurements were taken from distinct samples or whether the same sample was measured repeatedly
☐	☒	The statistical test(s) used AND whether they are one- or two-sided<br>*Only common tests should be described solely by name; describe more complex techniques in the Methods section.*
☒	☐	A description of all covariates tested
☒	☐	A description of any assumptions or corrections, such as tests of normality and adjustment for multiple comparisons
☐	☒	A full description of the statistical parameters including central tendency (e.g. means) or other basic estimates (e.g. regression coefficient) AND variation (e.g. standard deviation) or associated estimates of uncertainty (e.g. confidence intervals)
☐	☒	For null hypothesis testing, the test statistic (e.g. *F*, *t*, *r*) with confidence intervals, effect sizes, degrees of freedom and *P* value noted<br>*Give P values as exact values whenever suitable.*
☒	☐	For Bayesian analysis, information on the choice of priors and Markov chain Monte Carlo settings
☒	☐	For hierarchical and complex designs, identification of the appropriate level for tests and full reporting of outcomes
☒	☐	Estimates of effect sizes (e.g. Cohen's *d*, Pearson's *r*), indicating how they were calculated

Our web collection on statistics for biologists contains articles on many of the points above.

Software and code

Policy information about availability of computer code

Data collection	No software was used to collect the data.
Data analysis	1. The orthologs of Rf4 in the Poaceae family were identified for phylogenetic analysis of Rf4 using the tblastn program in the GenBank database (https://www.ncbi.nlm.nih.gov/).<br>2. The nucleotide sequences of Rf4 homologs were aligned with MAFFT v.7.475 and manually adjusted using MEGA X v.10.1.7.<br>3. Phylogenetic analysis was conducted using the maximum likelihood method with MEGA X v.10.1.7.

For manuscripts utilizing custom algorithms or software that are central to the research but not yet described in published literature, software must be made available to editors and reviewers. We strongly encourage code deposition in a community repository (e.g. GitHub). See the Nature Portfolio guidelines for submitting code & software for further information.

Data

Policy information about availability of data

All manuscripts must include a data availability statement. This statement should provide the following information, where applicable:

- Accession codes, unique identifiers, or web links for publicly available datasets
- A description of any restrictions on data availability
- For clinical datasets or third party data, please ensure that the statement adheres to our policy

All data supporting the findings in this study will be made available.

Human research participants

Policy information about studies involving human research participants and Sex and Gender in Research.

Reporting on sex and gender	We don't have related issues.
Population characteristics	We don't have related issues.
Recruitment	We don't have related issues.
Ethics oversight	We don't have related issues.

Note that full information on the approval of the study protocol must also be provided in the manuscript.

Field-specific reporting

Please select the one below that is the best fit for your research. If you are not sure, read the appropriate sections before making your selection.

- [x] Life sciences
- [ ] Behavioural & social sciences
- [ ] Ecological, evolutionary & environmental sciences

For a reference copy of the document with all sections, see nature.com/documents/nr-reporting-summary-flat.pdf

Life sciences study design

All studies must disclose on these points even when the disclosure is negative.

Sample size	Multiple independent transformants were obtained in the experiment and three lines were randomly selected for analysis. sample size were determined based on previous reports and best practices.
Data exclusions	No data were excluded from the analysis.
Replication	All attempts at replication were successful. To ensure reliable reproducibility: All transgenic lines were used at least three independent lines for further experiments analysis. Ten independent lines were used for pollen viability and seed setting rate calculate, respectively. qRT-PCR analysis data were calculated from three biological replicates.
Randomization	All Plants for genetic analysis were randomly allocated into experimental group.
Blinding	The samples were blindly processed during the experiment.

Reporting for specific materials, systems and methods

We require information from authors about some types of materials, experimental systems and methods used in many studies. Here, indicate whether each material, system or method listed is relevant to your study. If you are not sure if a list item applies to your research, read the appropriate section before selecting a response.

Materials & experimental systems

n/a	Involved in the study	
x		Antibodies
x		Eukaryotic cell lines
x		Palaeontology and archaeology
x		Animals and other organisms
x		Clinical data
x		Dual use research of concern

Methods

n/a	Involved in the study	
x		ChIP-seq
x		Flow cytometry
x		MRI-based neuroimaging
